# Chemosynthetic alphaproteobacterial diazotrophs reside in deep-sea cold-seep bottom waters

**DOI:** 10.1128/msystems.00176-24

**Published:** 2024-08-06

**Authors:** Jiawei Chen, Lixia Deng, Xiao Wang, Cheng Zhong, Xiaomin Xia, Hongbin Liu

**Affiliations:** 1 Department of Ocean Science, Hong Kong University of Science and Technology, Hong Kong, China; 2 College of Chemistry and Chemical Engineering, Southwest Petroleum University, Chengdu, Sichuan, China; 3 Key Laboratory of Tropical Marine Bio-resources and Ecology, South China Sea Institute of Oceanology, Chinese Academy of Sciences, Guangzhou, China; 4 Innovation Research Center for Carbon Neutralization, Fujian Key Laboratory of Marine Carbon Sequestration, Xiamen University, Xiamen, China; 5 Hong Kong Branch of Southern Marine Science and Engineering Guangdong Laboratory (Guangzhou), Hong Kong, China; University of Delhi, Delhi, India

**Keywords:** diazotroph, nitrogen fixation, chemosynthesis, cold seep, multi-omics, *Sagittula*

## Abstract

Nitrogen (N)-fixing organisms, also known as diazotrophs, play a crucial role in N-limited ecosystems by controlling the production of bioavailable N. The carbon-dominated cold-seep ecosystems are inherently N-limited, making them hotspots of N fixation. However, the knowledge of diazotrophs in cold-seep ecosystems is limited compared to other marine ecosystems. In this study, we used multi-omics to investigate the diversity and catabolism of diazotrophs in deep-sea cold-seep bottom waters. Our findings showed that the relative abundance of diazotrophs in the bacterial community reached its highest level in the cold-seep bottom waters compared to the cold-seep upper waters and non-seep bottom waters. Remarkably, more than 98% of metatranscriptomic reads aligned on diazotrophs in cold-seep bottom waters belonged to the genus *Sagittula*, an alphaproteobacterium. Its metagenome-assembled genome, named Seep-BW-D1, contained catalytic genes (*nifHDK*) for nitrogen fixation, and the *nifH* gene was actively transcribed *in situ*. Seep-BW-D1 also exhibited chemosynthetic capability to oxidize C1 compounds (methanol, formaldehyde, and formate) and thiosulfate (S_2_O_3_^2−^). In addition, we observed abundant transcripts mapped to genes involved in the transport systems for acetate, spermidine/putrescine, and pectin oligomers, suggesting that Seep-BW-D1 can utilize organics from the intermediates synthesized by methane-oxidizing microorganisms, decaying tissues from cold-seep benthic animals, and refractory pectin derived from upper photosynthetic ecosystems. Overall, our study corroborates that carbon-dominated cold-seep bottom waters select for diazotrophs and reveals the catabolism of a novel chemosynthetic alphaproteobacterial diazotroph in cold-seep bottom waters.

**IMPORTANCE:**

Bioavailable nitrogen (N) is a crucial element for cellular growth and division, and its production is controlled by diazotrophs. Marine diazotrophs contribute to nearly half of the global fixed N and perform N fixation in various marine ecosystems. While previous studies mainly focused on diazotrophs in the sunlit ocean and oxygen minimum zones, recent research has recognized cold-seep ecosystems as overlooked N-fixing hotspots because the seeping fluids in cold-seep ecosystems introduce abundant bioavailable carbon but little bioavailable N, making most cold seeps inherently N-limited. With thousands of cold-seep ecosystems detected at continental margins worldwide in the past decades, the significant role of cold seeps in marine N biogeochemical cycling is emphasized. However, the diazotrophs in cold-seep bottom waters remain poorly understood. Through multi-omics, this study identified a novel alphaproteobacterial chemoheterotroph belonging to *Sagittula* as one of the most active diazotrophs residing in cold-seep bottom waters and revealed its catabolism.

## INTRODUCTION

Bioavailable nitrogen (N), an essential element for cellular growth and division, is critical for biological productivity in marine ecosystems (1, 2). The production of bioavailable N is controlled by N-fixing organisms (i.e., diazotrophs) through the reduction of dinitrogen gas (N_2_) to ammonia (NH_3_), and the key enzymes catalyzing this process are nitrogenases encoded by the *nifH* and *nifDK* genes (3). Marine diazotrophs contribute to nearly half of the global fixed N (4) and perform N fixation in various marine ecosystems. While the ecological and biogeochemical importance of diazotrophs in the sunlit ocean and oxygen minimum zones (OMZs; the water column of several restricted regions of the ocean basins where there were low oxygen concentrations) has been well documented (5–7), recent studies have revealed the phylogenetic and catabolic diversity of diazotrophs in previously overlooked environments such as the deep-sea abyssal plain (8) and cold-seep sediments (9).

Cold seeps are extreme deep-sea environments where methane-rich fluids from subsurface reservoirs leak to the seafloor due to gravitational and tectonic forces. These seeping fluids introduce abundant bioavailable carbon (i.e., methane) but little bioavailable N into cold-seep ecosystems, resulting in most cold seeps being inherently N-limited and making them hotspots of N fixation (10). Over the past few decades, thousands of cold-seep systems have been detected at continental margins worldwide (11), highlighting the significant role of cold seeps in marine N biogeochemical cycling.

The key diazotrophs in cold-seep sediments are anaerobic methanotrophic archaea (ANME) and their sulfate-reducing bacterial partners (SRB) (9, 12). ANME-SRB consortia perform N fixation while anaerobically oxidizing methane and reducing sulfate. The capability of N fixation in ANME-SRB consortia has been demonstrated through NanoSIMS analysis (12, 13), and N fixation rates in cold-seep sediments are almost three times higher than those in background deep-sea sediments (14). Recently, multi-omics approaches have been used to investigate the diversity, distribution, and *in situ* activity of diazotrophs in cold-seep sediments (9), identifying phylogenetically diverse nitrogenase genes and expanding the diversity of cold-seep diazotrophic lineages. Although approximately 90% of the methane from deep marine sediments is consumed via anaerobic oxidation of methane (AOM) before reaching the seafloor (15), leaking methane in the water column can still reach up to 100 m above the seepage sites (16, 17). Methane seepage in bottom waters fuels free-living and symbiotic aerobic methane-consuming microbes, resulting in significantly higher benthic oxygen uptake at cold seeps than non-seeping seafloor (11). In addition, the seepage intensity strongly impacts the community structures of benthic animals and prokaryotes (18). With the continuous input of bioavailable carbon, cold-seep bottom waters can also be N-limited environments that select for diazotrophs. This raises the question of, in the cold seeps, whether the N fixation process is coupled with carbon-related chemosynthesis. However, little is known about the N fixation in cold-seep bottom waters compared with other marine ecosystems.

To address this knowledge gap, we investigated the phylogenetic and functional diversity of diazotrophs in the cold-seep bottom waters through metagenomics analysis. We also examined the *in situ* activity of diazotrophs through metatranscriptomic data. In addition, we compared diazotroph abundance and community among seep sites with different seepage activity, as well as samples from the euphotic and aphotic layers of the water column above the cold seeps, to elucidate key factors controlling diazotroph distribution and identify the niches for cold-seep diazotrophs. Our study highlights that deep-sea cold-seep bottom waters are overlooked hotspots of N fixation and provides insights into the functional adaptation of diazotrophs to cold-seep bottom waters.

## MATERIALS AND METHODS

### Sample collection and geochemical analysis

We conducted a research cruise at Haima cold seep (16°43′N, 110°28′E) in the South China Sea using R/V Haiyangdizhi VI in May 2022. Bottom waters (~1,400 m depth) were collected from four sites: three seep sites (i.e., high-intensity seepage [HS] site with mussel bed and continuous bubbling of methane gas, medium-intensity seepage [MS] site with live and dead mussels and live tubeworms, and low-intensity seepage [LS] site with clam bed and live tubeworms) and one control site (i.e., non-seepage [NS] site without any cold-seep-specific benthic animals and far from the three cold-seep sites). Water and sediment samples were collected using the remotely operated underwater vehicle (ROV) “Haima.” We also collected water samples using a “Sea-Bird 911” conductivity-temperature-depth (CTD; General Oceanics, Miami, FL, USA) rosette system from the euphotic (0, 50, and 100 m depth) and aphotic (600, 900, and 1,200 m depth) layers of the water column above the seep sites. For metagenomic samples, approximately 8 L of water samples was sequentially filtered onto 3-µm-pore and 0.22-µm-pore polycarbonate membranes (GVS, Roma, Italy) to collect particle-attached and free-living microbes, respectively. For metatranscriptomic samples, approximately 15 L of water samples was filtered onto 0.22-µm-pore polycarbonate membranes (GVS, Roma, Italy). Following filtration, the membranes were flash-frozen in liquid nitrogen immediately and stored at −80°C until further use.

To confirm the differences in seepage intensity among the three seep sites, we collected three push cores from each seep site using the ROV “Haima” for geochemical analysis. On board in a cold room at 4°C, subsamples of the water-sediment interface (0–2 cm surface sediment) were separated from the push cores, and the porewater of the water-sediment interface was extracted using Rhizon samplers (Rhizosphere Research Products, Wageningen, Netherlands). The concentrations of methane and sulfide were measured using Agilent 6850 Series II GC (Agilent, Santa Clara, CA, USA) and SmartChem200 Wet Chemistry Analyzer (KPM Analytics, Westborough, MA, USA), respectively. The stable carbon isotopic composition of DIC (δ13C-DIC) was measured using a Delta V Advantage mass spectrometer (Thermo Fisher Scientific, Poway, CA, USA) linked to a GasBench II device (Thermo Fisher Scientific, Poway, CA, USA). The GasBench II device was equipped with a PAL GC autosampler (CTC Analytics AG, Zwingen, Swizerland) and PoraPlotQ (30 m × 0.32 mm) GC Column (Agilent, Santa Clara, CA, USA). The mass spectrometer instrument was run at room temperature (25°C). Yielded CO_2_ was carried into the mass spectrometer with the aid of helium gas, and the δ13C value was measured. The helium flow is 0.5 mL/min, and the GC column is held at 70°C. For each sample, five replicates were sequentially injected, and the average value of the last three injections was recorded. The results are expressed in the standard delta (δ) notation per mil (‰). The δ13C values were relative to Vienna Pee Dee Belemnite (VPDB). Two carbonate standards, NBS-18 and IAEA-CO-8, were measured to determine the optimal extraction procedure.

### Nucleic acid extraction and sequencing

Total DNA and RNA were extracted using DNeasy PowerWater Kits (Qiagen, Hilden, Germany) and RNeasy Plus Kits (Qiagen, Hilden, Germany), respectively, according to the manufacturer’s protocol. DNA quality was measured using the Qubit dsDNA Assay Kit in a Qubit 2.0 Fluorometer (Thermo Fisher Scientific, Waltham, MA, USA). RNA quality and integrity were measured using a NanoDrop spectrophotometer (Thermo Fisher Scientific, MA, USA) and the RNA Nano 6000 assay kit in conjunction with the Agilent Bioanalyzer 2100 system (Agilent Technologies, CA, USA), respectively. Qualified DNA and RNA samples were assigned for metagenomic and metatrancriptomic sequencing using the NovaSeq 6000 system (Illumina, San Diego, CA, USA), and 150 bp paired-end reads were generated.

We collected 18 cold-seep bottom-water samples (four from each seepage site, six from the non-seepage site) and 36 water-column samples (12 from each seepage site) for DNA extraction (see Fig. S1 in the supplemental material). However, qualified DNA was only successfully extracted from some samples, and we eventually assigned 35 qualified DNA samples (16 from bottom waters, 12 from euphotic layers, and 7 from aphotic layers) for metagenomic sequencing (see Fig. S1 in the supplemental material). Due to the high demand for water samples and limited ROV diving opportunities, we could only collect water samples for RNA extraction in one seepage site. Therefore, only three qualified RNA samples from the MS site were assigned to metatranscriptomic sequencing.

### Profiling diazotroph relative abundance and community

The *nifH* gene has been commonly used as a marker to assess the distribution and community of diazotrophs (7, 19, 20). However, recent work by Mise et al. (21) has shown that approximately 20% of genomes that contain the *nifH* gene lack the *nifDK* genes, which encode essential subunits of nitrogenases (21). This suggests that *nifH* alone is not necessarily to be an indicator of diazotrophs. To address this issue, we defined a genome as a diazotroph only if it harbored all three nitrogenase genes (*nifHDK*) in this study.

To facilitate the identification of diazotrophs, we developed a pipeline called “Diaiden” (https://github.com/jchenek/Diaiden). In this pipeline, coding sequences (CDS) of genomes would be predicted using Prodigal v2.6.3 with the “-p meta” parameter (22). Then, CDS would be annotated using diamond v2.1.6 (23) with parameters “--sensitive -k 1 -e 1e-100 --id 50 --query-cover 75 --subject-cover 75” based on *nifHDK* sequences retrieved from the Kyoto Encyclopedia of Genes and Genomes (KEGG) database (24). Lastly, genomes would be identified as diazotroph genomes if the three catalytic genes (*nifHDK*) were detected. We applied the Diaiden pipeline to GTDB release R214, which comprises 85,205 prokaryotic genomes (25), resulting in 3,316 diazotrophs detected. We also collected the 48 diazotroph metagenome-assembled genomes (MAGs) recently recovered by Delmont et al. (7) from the global sunlit ocean (7) and customized a diazotroph database containing 3,364 genomes. Furthermore, we extracted *nifH* sequences from these diazotrophs and created a *nifH* database for subsequent analysis. In addition, to determine the abundance of prokaryotes in each sample, we developed a customized 16S ribosomal RNA database by removing chloroplast and mitochondria sequences from the SILVA 16S database v138 (26).

We employed Trimmomatic v0.39 (27) to trim the 35 metagenomic data. The resulting clean reads were aligned to the customized *nifH* and 16S databases using CoverM v0.6.1 (https://github.com/wwood/CoverM) under “contig” mode with parameters “--methods reads_per_base --min-read-percent-identity 95 --min-read-aligned-percent 75.” To represent the relative abundance of *nifH* sequences in the prokaryotic community of each sample, we normalized the reads per base value of *nifH* sequences by the reads per base value of 16S sequences [(reads per base of *nifH*/reads per base of 16S ) × 10^6^]. Furthermore, we transformed the reads per base value of each *nifH* sequence into transcripts per kilobase million (TPM) to represent the diazotroph community and visualized it using ggplot2 R package v.3.5.0 (28).

### Metagenomic assembly, annotation, and binning

We categorized the 35 metagenomic samples into three groups: bottom water (BW), euphotic layer (Euph), and aphotic layer (Aph). Clean reads from the same group were co-assembled using MEGAHIT v1.2.9 (29) with parameters “--k-min 27 --k-max 147 --k-step 12.” We predicted the CDS of each assembly using Prodigal v2.6.3 with the “-p meta” parameter (22) and annotated them against various databases, including the KEGG database (24), the carbohydrate-active enzymes database (CAZy) (30), the peptidase database (MEROPS) (31), the transporter classification database (TCDB) (32), TransportDB 2.0 (33), the universal protein knowledgebase (UniProt) (34), Pfam A (35), and Clusters of Orthologous Genes (COGs) (36) databases using diamond v2.1.6 (23) with parameters “--sensitive -k 1 -e 1e-20 --id 30 --query-cover 75 --subject-cover 75.” The CDS annotated as *nifH* (K02588) were extracted and assigned for further phylogenetic analysis.

The clean reads of 16 BW samples were aligned to their assembly using Bowtie2 v2.4.4 (37) with default settings to receive the coverage of contigs. Genomic binning was implemented using three programs, including MetaBAT2 v2.12.1 (38), MaxBin2 v2.2.7 (39), and CONCOCT v1.1.0 (40), with 1.5 kb as contig length cut-offs. Furthermore, raw MAGs were refined using the “bin_refinement” module of MetaWRAP v1.3 (41) and anvi’o v7.1 (42). The quality and taxonomic information of MAGs were obtained using CheckM v1.1.2 (43) and Genome Taxonomy Database Toolkit (GTDB-TK) v1.6.0 (44), respectively. Diazotroph MAGs were identified using the Diaiden pipeline as described above.

### Examining activity using metatranscriptomic data

We trimmed the metatranscriptomic data using Trimmomatic v0.39 (27). The clean data were aligned to MAGs recovered from the bottom-water assembly using Bowtie2 v2.4.4 (37) in “--very-sensitive-local” mode. We summarized the read counts based on the results (SAM files) from Bowtie2 using featureCounts v.2.0.0 (45) with the following parameters: “-M, -O, --fraction.” Raw counts were converted to reads per kilobase million (RPKM) using edgeR R package v.3.30.3 (46). We then converted the RPKM to the TPM value as described previously (47).

### Phylogenetic and average nucleotide identity analyses

Taxonomy information was assigned to the *nifH* sequences retrieved from BW, Euph, and Aph assemblies using VSEARCH v2.7.0 (48) with “--id 0.7 --query_cov 0.75” parameters based on the customized *nifH* database. In addition, the retrieved *nifH* sequences were aligned using MUSCLE v3.8.31 (49) and trimmed using trimAl v1.2 (50). Maximum likelihood phylogenetic analysis was implemented using IQ-TREE v1.6.12 (51) with the ultrafast bootstrap parameter “-bb 1000” (52), and the optimal phylogenetic model was selected by ProtTest v3.4.2 (53).

We inferred maximum-likelihood trees for MAGs based on the multiple sequence alignment of 120 bacterial marker proteins (54) using GTDB-Tk v1.6.0 (44). In brief, amino acid sequences of the genomes were predicted using Prodigal v2.6.3 (22) and then aligned to Pfam and TIGRfam hidden Markov models using HMMER v3.3 (http://hmmer.org/). The optimal phylogenetic model was then selected using ProtTest v3.4.2 (53), and a phylogenetic tree was constructed using IQ-TREE v1.6.12 (51) with the ultrafast bootstrap parameter “-bb 1000” (52). Average nucleotide identity (ANI) analysis was implemented using dRep v3.2.2 (55) with parameters “-comp 50 -con 10 P_ani 0.9 S_ani 0.98

### Weighted correlation network and statistical analyses

We implemented a weighted correlation network analysis (WGCNA) using the *WGCNA* v.1.71 R package (56) with a “signed” network type to identify potential correlations among microbes in cold-seep bottom waters. The input data matrix comprised the relative abundance of recovered MAGs in 16 bottom-water samples. Relative abundance was calculated using CoverM v0.6.1 (https://github.com/wwood/CoverM) under “genome” mode with parameters “--methods relative_abundance --min-read-percent-identity 97 --min-read-aligned-percent 75.” We calculated soft thresholds using the “pickSoftThreshold” function based on a weighted correlation matrix.

Shapiro-Wilk test was implemented using the “shapiro.test” function in R software (57) to test whether the data were normally distributed. We applied non-parametric tests using the “wilcox.test” to evaluate the differences among groups with abnormal distribution, and the “t.test” to evaluate the differences among groups with normal distribution.

## RESULTS AND DISCUSSION

### Diazotroph relative abundance and community in cold-seep bottom waters

**Fig 1:**
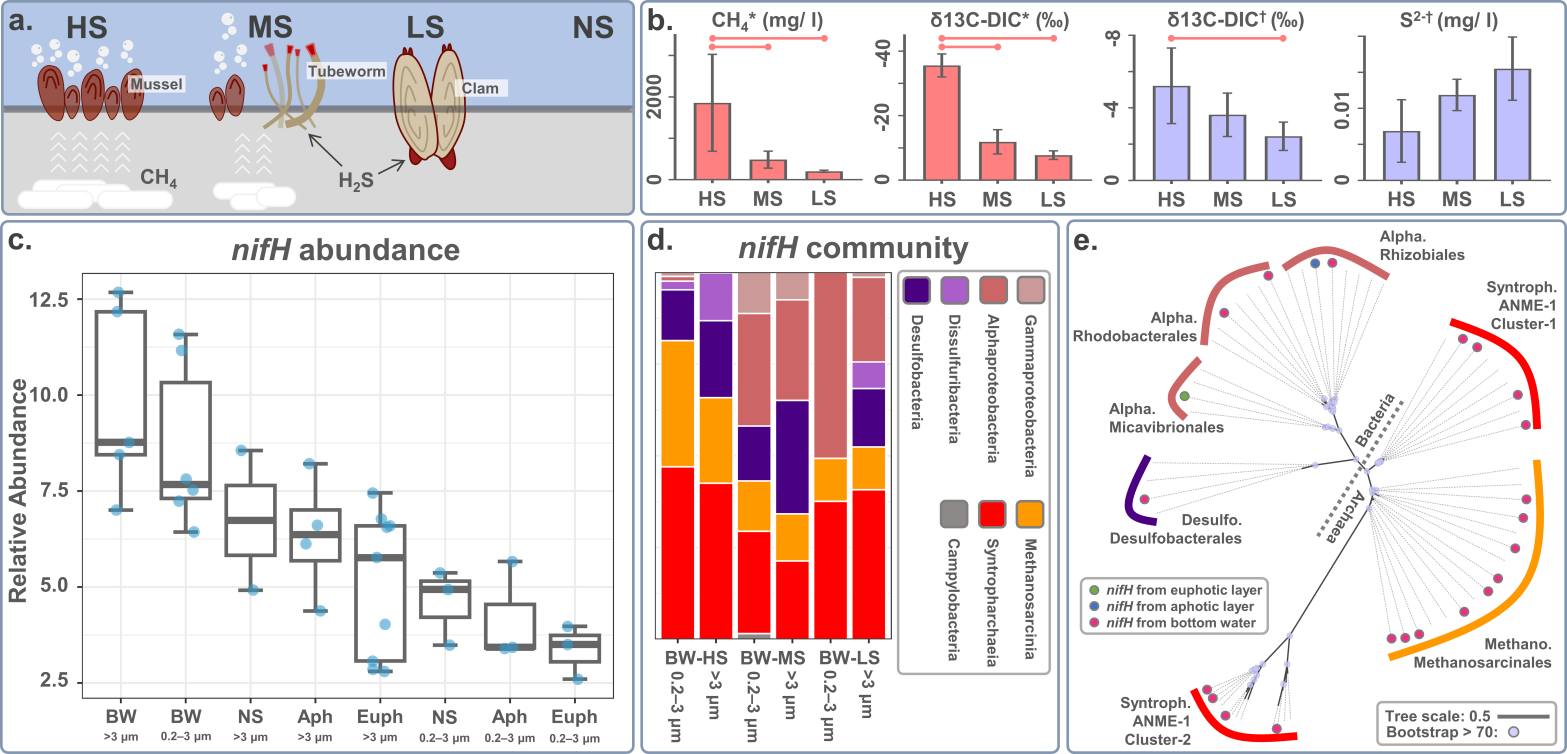
(a) Schematic image of the sampling sites. HS, high-intensity seepage site; MS, medium-intensity seepage site; LS, low-intensity seepage site. (b) Environmental factors of sampling sites, including methane (CH_4_), δ^13^C values of DIC (δ^13^C_DIC_), and sulfide (S^2-^). The red line indicates *P*-value (between-group differences) <0.05. *, porewater sample from the water-sediment interface; †, bottom-water sample. (c) Relative abundance of *nifH* sequences in different environments. The relative abundance was calculated via log2[(reads per base of *nifH*/reads per base of 16S ) × 10^6^]. BW, bottom waters; NS, non-seepage site; Euph, euphotic layer; Aph, aphotic layer. (d) The *nifH* community among seep sites with different seepage activity. (e) Maximum likelihood tree for the *nifH* sequence. Alpha., Alphaproteobacteria; Syntroph., Syntropharchaeia; Methano., Methanosarcinia; Desulfo., Desulfobacteria.

We collected water samples from three sites with varying seepage activities in the Haima cold seep (Fig. 1a). At the HS site, mussel beds and continuous gas bubbling were frequently observed on the seafloor. At the MS site, both live and dead mussels were present, and only a few gas-bubbling points were observed. The LS site had no live mussel, being dominated by clams, with no observed gas bubbling points. *In situ* images of the cold-seep landscapes can be found in our previous work (58, 59). Methane concentrations in the water-sediment interface showed a transparent gradient among the three seepage sites (HS site: 1857.6 ± 1169.3 mg/L; MS site: 484.6 ± 204.9 mg/L; and LS site: 203.1 ± 24.9 mg/L), consistent with the δ13C-DIC values in the water-sediment interface (HS site: −35.6 ± 3.5‰; MS site: −11.9 ± 3.8‰; and LS site: −7.8 ± 1.3‰, VPDB) and bottom waters (HS site: −5.2 ± 2.1‰; MS site: −3.6 ± 1.2‰; and LS site: −2.4 ± 0.8‰, VPDB), indicating that HS site had significantly higher methane-oxidizing activity than the MS and LS sites (*P*-value < 0.05) (Fig. 1b). Overall, both the landscapes and environmental factors indicated that the three sampling sites could be distinguished by their seepage activities.

In cold-seep sites, we compared the bottom waters (BW; *n* = 11) with the euphotic (Euph; *n* = 12) and aphotic (Aph; *n* = 7) layers. In addition, we also compared cold-seep bottom waters (BW;, *n* = 11) with bottom waters in non-seep sites (NS; *n* = 5). Our results showed that cold-seep bottom waters had the highest relative abundance of diazotrophs in the prokaryotic community compared with other water layers. (*P*-value < 0.05) (Fig. 1c; see Fig. S2a in the supplemental material). In addition, the relative abundance of diazotrophs was significantly higher in the HS site than in the MS and LS sites (*P*-value < 0.05) (see Fig. S2b in the supplemental material). These findings support the hypothesis that carbon-dominated cold-seep environments select for diazotrophs.

The diazotroph communities in cold-seep bottom waters were distinct from those in the sunlit ocean. One of the most notable differences was that the archaeal classes Methanosarcinia and Syntropharchaeia, which are absent in the global sunlit ocean (7), were prevalent in cold-seep bottom waters (Fig. 1d). In addition, Gammaproteobacteria mostly dominated the diazotroph communities in the global surface ocean (7, 60), while the predominant bacterial diazotroph in cold-seep bottom waters belonged to Alphaproteobacteria (Fig. 1d). Seepage activity also affected the diazotroph communities in cold-seep bottom waters. For example, Alphaproteobacteria only predominated the diazotroph community in the MS and LS sites (contributing to ~1% in the HS site, 27%–30% in the MS site, and 23%–51% in the LS site), while the abundance of archaeal diazotrophs was highest in the HS site (contributing to 66%–81% in the HS site, 34%–42% in the MS site, and 49%–52% in the LS site).

For phylogenetic analysis, we retrieved *nifH* sequences from the three metagenomic assemblies, including one from euphotic layers, one from aphotic layers, and 21 from bottom waters. The phylogenetic tree (Fig. 1e) showed that among the 21 *nifH* sequences from bottom waters, 17 belonged to archaea, 3 belonged to Alphaproteobacteria, and 1 belonged to Desulfobacteria. The 17 archaeal *nifH* sequences were affiliated with two orders, namely ANME-1 and Methanosarcinales (ANME-2 cluster archaea). Interestingly, the ANME-1 *nifH* sequences were grouped into two separate clusters. Cluster-1 was closely related to ANME-2 *nifH* sequences, while cluster-2 was distant from all other *nifH* sequences, indicating that the *nifH* genes in ANME-1 might have different evolutionary origins. The *nifH* gene of Desulfobacteria showed high similarity to the *nifH* of strain ETH-SRB1 (61), which frequently forms consortia with ANME. These ANME-SRB consortia are active diazotrophs in cold-seep sediments (9, 12–14), and their potential roles in oxic cold-seep bottom waters will be discussed below. The three alphaproteobacterial *nifH* sequences were affiliated with the genera *Bradyrhizobium*, *Sagittula*, and *Salipiger*. Species from *Bradyrhizobium* are well-known symbiotic nitrogen-fixing bacteria associated with plants (62). For the genus *Sagittula*, N-fixation capability has been reported in two strains, namely P11 (63) and MA-2 (64). P11 was isolated from the OMZs off Peru, and MA-2 was isolated from a coastal marine bacterial consortium in which gentisic acid was the sole carbon and energy source. *Salipiger* strains have been isolated from deep-sea waters (65) and mangrove sediment (66), but their N-fixation capability has been less studied.

### Reconstruction of diazotroph MAGs from cold-seep bottom waters

**Fig 2:**
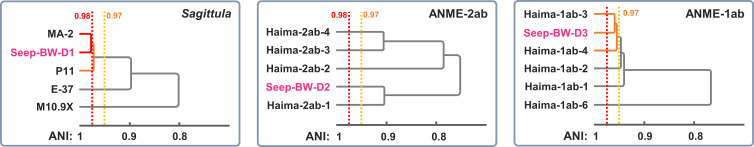
Average nucleotide identity dendrogram among genomes belonging to *Sagittula*, ANME-1ab, and ANME-2ab.

A total of 250 medium- to high-quality (completeness ≥ 50%, contamination < 10%) MAGs were recovered from cold-seep bottom waters, and three of them contained the *nifH* gene. Based on GTDB-TK and ANI analyses (Fig. 2), the three MAGs, Seep-BW-D1, Seep-BW-D2, and Seep-BW-D3, were affiliated with the genera *Sagittula* (Alphaproteobacteria), *Methanomarinus* (ANME-2), and *QENJ01* (ANME-1), respectively (see Table S1 in the supplemental material). Only Seep-BW-D1 (completeness: 86.79%, contamination: 0.99%) contained the three *nifHDK* nitrogenase genes, while Seep-BW-D2 (completeness: 86.6%, contamination: 0.33%) contained *nifHD* genes, and Seep-BW-D3 (completeness: 76.14%, contamination: 0%) only contained the *nifH* gene. To investigate their *in situ* activity, we aligned the three MAGs with metatranscriptomic reads. Among the 438,971 aligned metatranscriptomic reads, 432,374 (98.5%) were aligned to Seep-BW-D1, 1,762 (0.4%) were aligned to Seep-BW-D2, and 4,835 (1.1%) were aligned to Seep-BW-D3, indicating that the Alphaproteobacteria Seep-BW-D1 is the only active diazotroph in the cold-seep bottom waters detected in our studies. The annotation results of Seep-BW-D1 are shown in Table S2 in the supplemental material.

Multiple lines of evidence have demonstrated that ANME are active N-fixers in cold-seep sediments (9, 12–14). However, ANME are strict anaerobes whose activity is inhibited in the presence of oxygen (67). The oxygen concentration of bottom waters in the Haima cold seep was approximately 103–109 mM (18), making it an oxic habitat only suitable for aerobes. We compared the ANI among the ANME MAGs retrieved from both the bottom waters and the sediments of identical cold-seep sites (68) and observed a high degree of similarity among these ANME MAGs (Fig. 2). Therefore, the ANME in bottom waters were likely sourced from surface sediments and/or water-sediment interfaces due to water current disturbance and fluids associated with methane seepage. Since ANME were not active in cold-seep bottom waters and the catabolism of ANME has been well documented previously (9, 68), we would not further discuss Seep-BW-D2 and Seep-BW-D3 in this study.

### Comparative genomic analysis among alphaproteobacterial diazotrophs in ocean

The first *Sagittula* strain, E-37, was discovered from a coastal marine bacterial consortium in 1997 (69) and sequenced in 2018 (70). This strain was characterized by its lignin-degrading ability. In 2018, the first complete genome of *Sagittula* was obtained from strain P11, a diazotroph isolated from OMZs off Peru (63). Recently, another *Sagittula* strain, MA-2, was isolated from a coastal marine bacterial consortium. This strain grows on gentisic acid as the sole carbon and energy source, and its complete genome was successfully sequenced (64). So far, the Seep-BW-D1 recovered in this study was the only *Sagittula* genome obtained from deep-sea waters. Based on ANI analysis, the three *Sagittula* diazotrophs, Seep-BW-D1, MA-2, and P11, were found to be affiliated with the same subspecies due to their high nucleotide similarity (ANI ≥97%) (Fig. 2).

**Fig 3:**
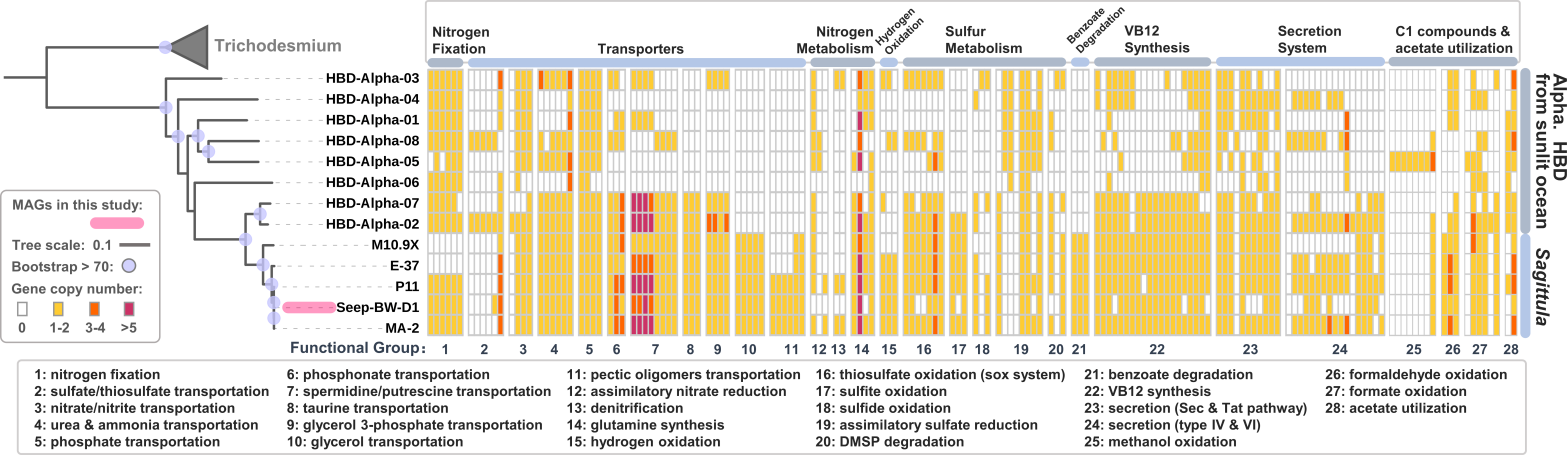
Comparison of lineage-specific functions associated with environmental adaptation in oceanic alphaproteobacterial diazotrophs and close relatives of *Sagittula*. The maximum likelihood tree is constructed based on a multiple sequence alignment of 120 bacterial single-copy marker proteins. Alpha., Alphaproteobacteria.

In addition to *Sagittula* relatives, we collected eight alphaproteobacterial heterotrophic bacterial diazotrophs (HBDs) recovered from the global sunlit ocean (7) and conducted comparative genomic analyses among them. The phylogenetic tree showed that none of the eight alphaproteobacterial HBDs from the sunlit ocean were affiliated with the genus *Sagittula* (Fig. 3; see Table S3 in the supplemental material). This indicates that the *Sagittula* diazotroph has distinct genomic adaptations that facilitate its predominance in deep-sea cold-seep bottom waters. The closest relative HBDs to *Sagittula* were HBD-Alpha-02 and HBD-Alpha-07, both belonging to the genus *Marinibacterium*. In addition, both *Sagittula* and *Marinibacterium* belong to the family Rhodobacteraceae.

**Fig 4:**
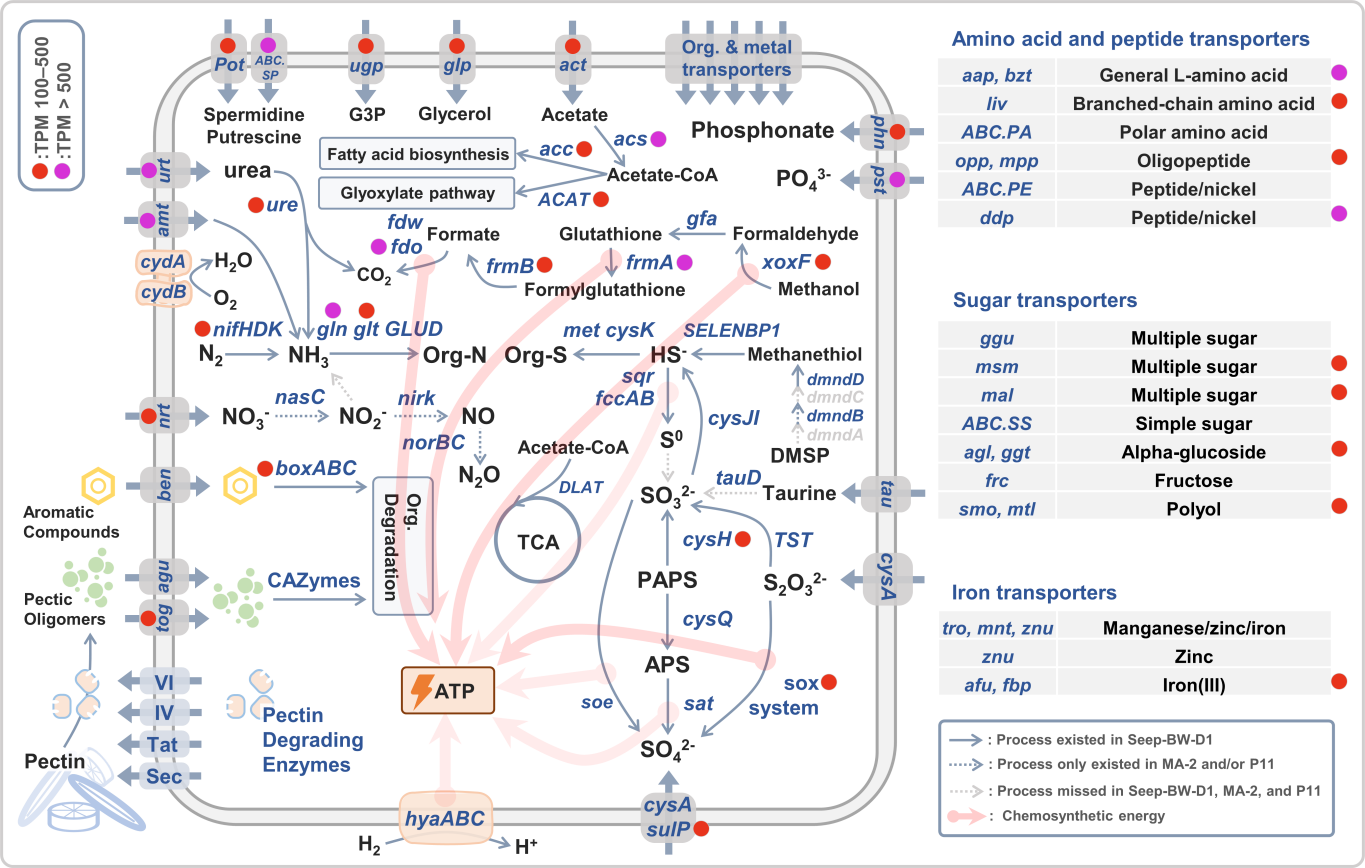
Schematic representation of the metabolic capacities and activities of Seep-BW-D1.

One remarkable genomic feature *Sagittula* diazotrophs shared was the ability to perform chemosynthesis (Fig. 3 and 4). *Sagittula* diazotroph genomes encoded enzymes for oxidizing various C_1_ compounds (methanol, formaldehyde, and formate), including lanthanide-dependent methanol dehydrogenase (*xoxF*) for the oxidation of methanol, S-(hydroxymethyl)glutathione synthase (*gfa*) for the oxidation of formaldehyde, and formate dehydrogenase (*fdo* and *fdw*) for the oxidation of formate. *Sagittula* diazotroph genomes also encoded enzymes involved in oxidizing reduced sulfur compounds (H_2_S, S_2_O_3_^2−^, and SO_3_^2−^), including the sox enzyme complex (*soxABCDXYZ*) for oxidizing thiosulfate (S_2_O_3_^2−^) to sulfate (SO_4_^2-^); sulfide:quinone oxidoreductase (*sqr*), cytochrome subunit of sulfide dehydrogenase (*fccA*), and sulfide dehydrogenase (*fccB*), mediating the oxidation of sulfide (HS^−^) to elemental sulfur (S^0^); and sulfite dehydrogenase (*soeABC*), mediating the oxidation of sulfite (SO_3_^2−^) to sulfate (SO_4_^2−^). In addition, *Sagittula* diazotroph genomes encoded Ni, Fe hydrogenase (*hyaABC*) for H_2_ oxidation. Hydrogenase could facilitate N fixation in aerobic organisms by acting as an oxygen scavenger to protect nitrogenase from oxygen inhibition, preventing the inhibition of N_2_ reduction by H_2_ generated by nitrogenase, and recycling H_2_ produced by nitrogenase to provide reducing power (71). Considering that cold seeps are typical chemosynthetic ecosystems, *Sagittula* diazotrophs could benefit from the chemical energy derived from cold seeps *via* their chemosynthetic capability.

**Fig 5:**
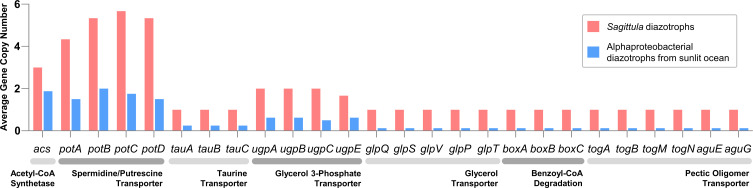
Average copy number of genes among *Sagittula* diazotrophs and alphaproteobacterial diazotrophs from the sunlit ocean.

We examined the genomic potential for inorganic carbon (CO_2_) fixation in alphaproteobacterial diazotrophs. Our results showed that most of the tested alphaproteobacterial diazotrophs did not contain genes encoding enzymes for CO_2_ fixation, except for HBD-Alpha-08, which encoded ribulose-bisphosphate carboxylase (*rbcL*) involved in Calvin-Benson cycle. Therefore, the source of organic carbon is crucial for most alphaproteobacterial diazotrophs. Acetate is a key organic carbon source in marine waters and sediments (72, 73). Since cold-seep sediments contain abundant acetate exported by methane-oxidizing microorganisms that potentially sustain microbial communities (74), the surface sediments and the water-sediment interface can be sources of bottom-water acetate. Our results showed that, compared with other alphaproteobacterial diazotrophs, *Sagittula* diazotrophs contained a higher copy number of genes encoding acetyl-CoA synthetase (*acs*) that convert acetate into acetate-CoA (Fig. 5; see Table S3 in the supplemental material). This result indicates that *Sagittula* diazotrophs may better utilize acetate than alphaproteobacterial diazotrophs residing in the sunlit oceans. In addition, benthic animals can be important organic carbon sources in cold-seep bottom waters. Organic compounds, such as putrescine, spermidine, taurine, glycerol 3-phosphate, and glycerol, can be released into water environments from decaying animal tissues. Our results showed that *Sagittula* diazotrophs distinctly encoded high-affinity transport systems to uptake these compounds, including spermidine/putrescine transporter (*potABCD*), taurine transporter (*tauABC*), glycerol 3-phosphate transporter (*upgABCE*), and glycerol transporter (*glpQSVPT*) (Fig. 5; see Table S3 in the supplemental material). Moreover, deep-sea cold-seep ecosystems can also receive organic compounds from the upper ecosystem relying on photosynthesis (75). The main organic compounds reaching deep-sea seafloor are refractory organics, such as lignin, pectin, and aromatics (76, 77). Our results showed that *Sagittula* diazotrophs encoded extra genes encoding proteins involving benzoyl-CoA degradation (*boxABC*) and pectic oligomer transportation (*togABMN* and *aguEG*) (Fig. 5; see Table S3 in the supplemental material). Overall, compared with other alphaproteobacterial diazotrophs, *Sagittula* diazotrophs have a higher potential to utilize kinds of organic compounds derived from methane-oxidizing microorganisms, cold-seep benthic animals, and refractory organics from surface waters.

### Transcriptional activity of Seep-BW-D1 in cold-seep bottom waters

We aligned metatranscriptomic reads to the Seep-BW-D1 genome to examine its transcriptional activity in cold-seep bottom waters (Fig. 4; see Table S4 in the supplemental material). Our results showed that the *nifH* gene was actively expressed in Seep-BW-D1, indicating that Seep-BW-D1 can fix nitrogen *in situ*. The nitrogenase encoded by *nifHDK* carries an iron-molybdenum cofactor (FeMo-co), which is one of the most complex metal cofactors known to date (3). Genes involved in FeMo-co biosynthesis, including *nifENB*, were encoded by the Seep-BW-D1 genome. The Fe and Mo can be limited factors controlling N fixation in the oligotrophic open ocean, but the limitation can be mitigated in cold-seep bottom waters. This is because cold-seep sediments are rich in Mo and Fe, and substantial amounts of metals can be released into cold-seep bottom waters through seeping fluids (78, 79). Each FeMo-co contains one Mo and seven Fe atoms, indicating a higher demand for Fe than Mo in diazotrophs. Our results showed that the iron transporters, including *afu* and *fbp*, were actively expressed in Seep-BW-D1, which may be due to the high cellular Fe requirements. The activity of nitrogenase is inhibited under high intracellular oxygen levels (80). Seep-BW-D1 contained and expressed the gene encoding cytochrome bd terminal oxidase (*cydA*) (Fig. 4), which can decrease intracellular oxygen levels through uncoupled respiration and protect nitrogenase from oxygen (81). Hence, as revealed by the MAG and metatranscriptome, Seep-BW-D1 is genetically capable of fixing nitrogen with respiratory protection in oxygenated cold-seep bottom waters.

We found that various genes for N uptake, including *urt* for urea, *amt* for ammonia, *nrt* for nitrate/nitrite, *aap* and *liv* for amino acids, and *app* and *ddp* for peptides, were actively expressed, indicating that Seep-BW-D1 had multiple N sources to fulfill its N demand. In addition, the expression level of *urt* gene was 5–10 times higher than any other N transporter, indicating urea is a preferable organic N source for Seep-BW-D1 in cold-seep bottom waters. As a diazotroph, phosphorus can be a critical limited element for Seep-BW-D1 (2). Our results showed that phosphate transporter gene *pst* and phosphonate transporter gene *phn* were actively expressed, indicating that Seep-BW-D1 can utilize both inorganic and organic phosphorus to fulfill its phosphate demand.

Although Seep-BW-D1 contained genes for oxidizing various reduced compounds, not all were expressed activity. Our results showed that the most active chemotrophic process was the oxidation of formate catalyzed by *fdo*, followed by the oxidation of methanol (catalyzed by *xoxF*) and thiosulfate (catalyzed by sox system) (Fig. 4; see Table S4 in the supplemental material). All three compounds can be biogenetic by microbes through methane-oxidizing processes. For example, formate is one of the key intermediate compounds between ANME and SRB in the AOM process (82); methanol can be synthesized by methanotrophs in the water-sediment interface through aerobically methane oxidation (83); and thiosulfate can be a by-product of sulfate reduction coupled with AOM (82). These findings suggest that although Seep-BW-D1 cannot obtain energy from methane directly, its primary energy sources are still derived from methane, indicating that cold-seep ecosystems are ideal habitats for *Sagittula* diazotroph Seep-BW-D1.

In addition to the energy sources, we also investigated the carbon sources of Seep-BW-D1. We found that abundant transcripts in the metatranscriptome were mapped to genes involved in acetate utilization (Fig. 4). Although acetate was reported to be microbial energy and carbon source in water column (72), we only found genes for the assimilation but not oxidation of acetate highly expressed in Seep-BW-D1, including acetyl-CoA C-acetyltransferase (*ACAT*) involved in glyoxylate pathway and acetyl-CoA carboxylase (*acc*) for fatty acid biosynthesis. Therefore, acetate is an important organic carbon source but not an energy source for Seep-BW-D1. In addition, many organic carbon transporters were found to be highly expressed, including transport systems for spermidine/putrescine (*Pot* and *ABC.SP*), glycerol 3-phosphate transporter (*ugp*), glycerol (*glp*), oligogalacturonide (encoded by *tog*), and multiple sugars (*msm* and *mal*). Moreover, we screened the activity of carbohydrate-active enzymes (CAZymes) and peptidases (see Fig. S3 in the supplemental material). Gene expression profiles showed that some CAZymes were highly active Seep-BW-D1, including GH102, GH103, and GH23, involved in the degradation of peptidoglycans. The most active peptidase family was C26 (gamma-glutamyl hydrolase), involving the turnover of folyl poly-gamma-glutamates. In general, *Sagittula* diazotroph Seep-BW-D1 actively utilized kinds of organic compounds derived from methane-oxidizing microorganisms, cold-seep benthic animals, and refractory organics from surface waters.

### Potential interactions between Seep-BW-D1 and its co-occurring microbes

Aggregate formation may be one behavioral strategy enabling diazotrophs to generate a low oxygen-level environment (84). Many diazotrophs can form aggregation and perform co-evolutionary mechanisms with their associated organisms (85, 86). Based on the black queen hypothesis (87), certain functions or products of diazotrophs can be “leaky,” which affect or be used by associated organisms, and are therefore considered “public goods.” Associated organisms that use these public goods may then experience positive selective pressure resulting in the loss of their costly pathways that are responsible for those “public goods.” *Sagittula* strain P11, a close relative of Seep-BW-D1, was observed to form aggregates and exhibited a complex relationship with its associated microbes (63). We also identified genes involved in aggregation formation in Seep-BW-D1, including genes encoding secretion systems (88) and extracellular polysaccharides synthesis (89) (see Table S2 in the supplemental material), suggesting Seep-BW-D1 could exhibit close interactions with its associated microbes.

We applied WGCNA analysis and found that Seep-BW-D1 co-occurred with MAGs from the module ME-Blue (see Fig. S4a in the supplemental material). MAGs from this module belonged to different taxonomies, including phylum Proteobacteria, Verrucomicrobiota, Myxococcota, Planctomycetota, and Actinobacteriota (see Table S5 in the supplemental material). We selected 11 medium- to high-quality MAGs (completeness > 80%) from ME-Blue and applied comparative genomic analysis to identify the potential “public goods” and the lost costly pathways (see Fig. S4b and c in the supplemental material). Our results showed that none of these MAGs could synthesize vitamin B12 (VB12), which is crucial for cell growth, while Seep-BW-D1 contained and expressed genes involved in the whole process of VB12 synthesis. By contrast, Seep-BW-D1 did not have the gene *tauD* for the last step of taurine utilization, while its associated microbes from ME-Blue contained genes encoding this enzyme. Moreover, the associated microbes from ME-Blue encoded various enzymes for pectin degrading but did not encode pectin oligomers transporters, while Seep-BW-D1 distinctly contained pectin oligomers transport system. In general, we present molecular evidence that Seep-BW-D1 may be closely associated with some microbes in cold-seep bottom waters, and they might maintain their relationships via sharing “public goods” such as VB12 and kinds of enzymes.

### Identifying the niche of Seep-BW-D1

**Fig 6:**
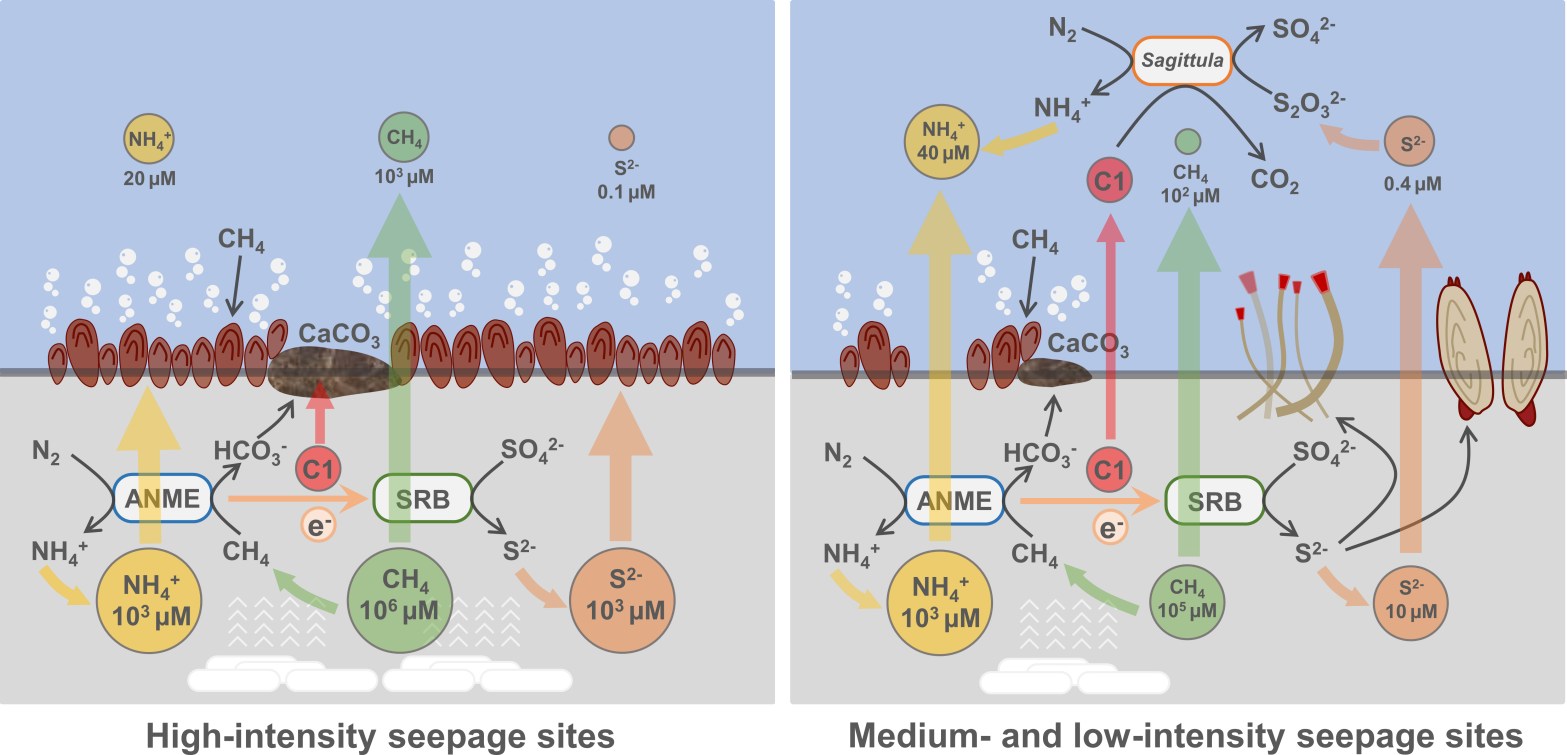
Schematic representation of the niche of alphaproteobacterial diazotroph *Sagittula* in cold-seep bottom waters.

We observed niche partitioning across seepage activity among diazotrophs in cold-seep bottom waters. The methane-oxidizing diazotroph ANME dominated in the HS site. By contrast, the sulfur-oxidizing diazotroph *Sagittula* was more predominant in the MS and LS seepage sites (Fig. 1d). The heterogeneity of energy sources may explain this distribution pattern. HS site has a significantly higher methane concentration (~10^6^ µM in sediment, ~10^3^ µM in water) than MS and LS sites (~10^5^ µM in sediment, ~10^2^ µM in water) sites (Fig. 1b and 6) (18), which benefits the prevalence of methane-oxidizing diazotrophs. By contrast, the H_2_S concentration is higher in the bottom waters of MS and LS sites (~0.4 µM) than in the HS site (~0.1 µM) (Fig. 1b and 6), which facilitates the prevalence of sulfur-oxidizing diazotrophs. Considering that the H_2_S in cold seeps are mainly synthesized in the sulfate-methane transition zone of sediments coupled with AOM, it is intriguing that H_2_S concentration is higher in MS and LS than in HS bottom waters. In fact, not only H_2_S concentration but other inorganic nutrients, such as nitrate, nitrite, ammonium, and phosphate, also have a higher concentration at LS than at HS sites (18). Considering these compounds possibly sourced from deep fluids (90), we hypothesize that the crowded mussel bed and authigenic carbonates in the HS site block the surface seafloor and thus reduce the upwelling of H_2_S and C1 compounds. In the MS and LS sites, however, the biogenetic barrier is decreased because of the lower methane concentration. In addition, the predominant benthic animals, such as clams and tubeworms, would dig into deep sediment and uptake H_2_S through their foot or roots for chemosynthesis (91, 92). With less biogenetic barrier and more robust animal behavior, the MS and LS sediments would likely release more H_2_S and C1 compounds to the bottom waters, making this environment selected for the sulfur-oxidizing diazotrophs Seep-BW-D1 (Fig. 6).

Sharp geochemical and redox gradients persist in cold-seep sediments and waters. For example, the H_2_S concentration in the anoxic cold-seep sediments is 10^2^–10^4^ times higher than in the oxic cold-seep bottom waters. By contrast, C1 compounds, such as methanol, are more stable in the oxic environment, whose concentration in seawater (up to 429 nM) is similar to sediment (up to 112 nM) (93). Therefore, with the increased distance from the seepage site, the chemosynthetic diazotroph *Sagittula* may rely more on C1 compounds than reduced sulfur compounds. Nevertheless, since both C1 and sulfur compounds in cold seep are sourced from the methane-oxidizing process, and methane gas can reach up to 100 m above the seepage site (16, 17), Seep-BW-D1 may restrict its distribution in the bottom waters within the methane seeping region.

### Conclusions

In this study, we found that the relative abundance of diazotrophs in the bacterial community reached its highest level in the cold-seep bottom waters compared to the cold-seep upper waters and non-seep bottom waters, corroborating that the carbon-dominated cold-seep environments are hotspots of N fixation. Moreover, our results showed that the most active diazotroph in cold-seep bottom waters is an Alphaproteobacterium belonging to the genus *Sagittula*, named Seep-BW-D1.

To address the N limitation in cold seeps, Seep-BW-D1 adopted the capability to fix inorganic N and assimilate organic N. As a diazotroph, seep-BW-D1 contained catalytic genes (*nifHDK*) and biosynthetic genes (*nifENB*) for nitrogen fixation, and its nitrogenase-encoding genes were transcribed actively *in situ*. Moreover, Seep-BW-D1 expressed transport systems for various organic N, and its preferred organic N was urea. For carbon source, although Seep-BW-D1 cannot fix inorganic carbon, it can assimilate various kinds of organic carbon that are abundant in cold-seep ecosystems, including acetate synthesized by methane-oxidizing microorganisms, spermidine/putrescine from the decaying tissues of cold-seep benthic animals, and refractory pectin from upper photosynthetic ecosystems. Seep-BW-D1 exhibited chemosynthetic capability and actively oxidized methane-derived compounds, such as C1 compounds (methanol, formaldehyde, and formate) and thiosulfate (S_2_O_3_^2−^). Seep-BW-D1 was more abundant in MS and LS sites than in HS sites. This may be because the less biogenetic barrier and more robust animal behavior in the MS and LS sites facilitate the release of C1 and reduced sulfur compounds into bottom waters, which benefit the growth of Seep-BW-D1.

In general, we corroborate that the carbon-dominated cold-seep bottom waters select for diazotrophs and reveal the ecological functions and metabolic strategies of a novel chemosynthetic N-fixing *Sagittula* in cold-seep bottom waters.

## Data Availability

Metagenomic sequences have been deposited in the National Center for Biotechnology Information (NCBI) under the BioProject number PRJNA1073185. Metatranscriptomic sequences have been deposited in NCBI under the BioProject number PRJNA1073186. Three diazotroph MAGs recovered in this study have been deposited in the National Omics Data Encyclopedia (NODE) under the project number OEP005056. The 250 MAGs recovered from cold-seep bottom waters have been deposited in figshare (https://doi.org/10.6084/m9.figshare.25975903.v1). The "Diaiden" pipeline is available on GitHub (https://github.com/jchenek/Diaiden). Authors declare that all data supporting the findings of this study are available within the article and its supplemental information files or from the corresponding authors upon request.

## SUPPLEMENTAL MATERIAL

Fig. S1 msystems.00176-24-s0001.tif Schematic representation of sampling sites.

Fig. S2 msystems.00176-24-s0002.tif Relative abundances of nifH sequences.

Fig. S3 msystems.00176-24-s0003.tif Expression profiles of CAZymes and peptidases in Seep-BW-D1.

Fig. S4 msystems.00176-24-s0004.tif WGCNA network analysis, potential interactions between Seep-BW-D1 and its co-occurring microbes, and copy number of genes involved in different functional categories among genomes in ME-Blue.

Table S1 msystems.00176-24-s0005.xlsx Genomic features of Seep-BW-D1, Seep-BW-D2, and Seep-BW-D3.

Table S2 msystems.00176-24-s0006.xlsx Annotation results of Seep-BW-D1 based on various databases.

Table S3 msystems.00176-24-s0007.xlsx The copy number of genes associated with environmental adaptation in oceanic alphaproteobacterial diazotrophs and close relatives of Sagittula.

Table S4 msystems.00176-24-s0008.xlsx Transcriptional activity of Seep-BW-D1 in cold-seep bottom waters.

Table S5 msystems.00176-24-s0009.xlsx Genomic information of MAGs co-occurred with Seep-BW-D1.

ASM does not own the copyrights to Supplemental Material that may be linked to, or accessed through, an article. The authors have granted ASM a non-exclusive, world-wide license to publish the Supplemental Material files. Please contact the corresponding author directly for reuse.
