# Supplementary figures and images for: Chemosynthetic alphaproteobacterial diazotrophs reside in deep-sea cold-seep bottom waters
Source: mSystems. 2024 Aug 6;9(9):e00176-24. doi: 10.1128/msystems.00176-24 (PMC11406894; doi:10.1128/msystems.00176-24)

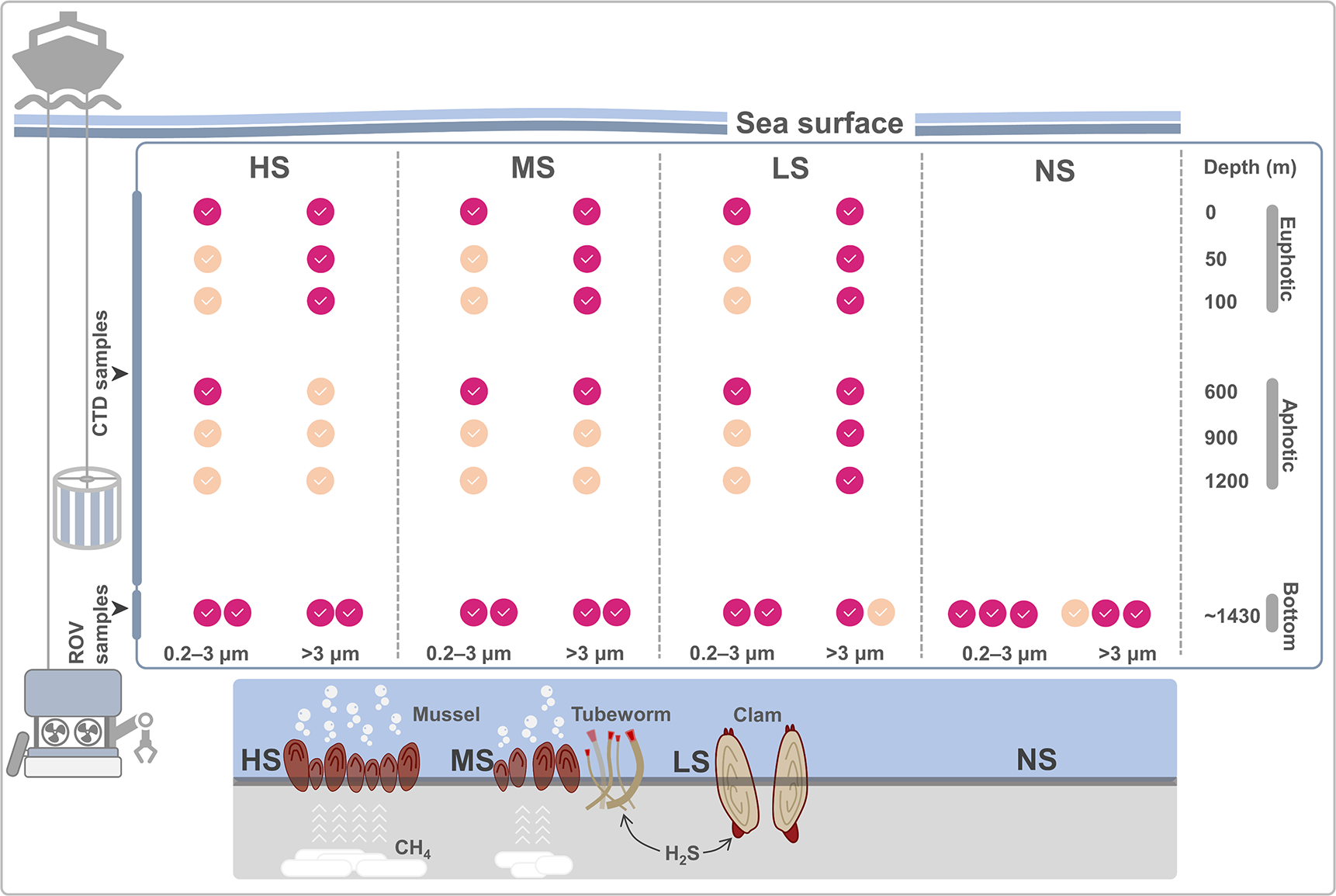

Supplement: Fig. S1 — Schematic representation of sampling sites. [file msystems.00176-24-s0001.tif]

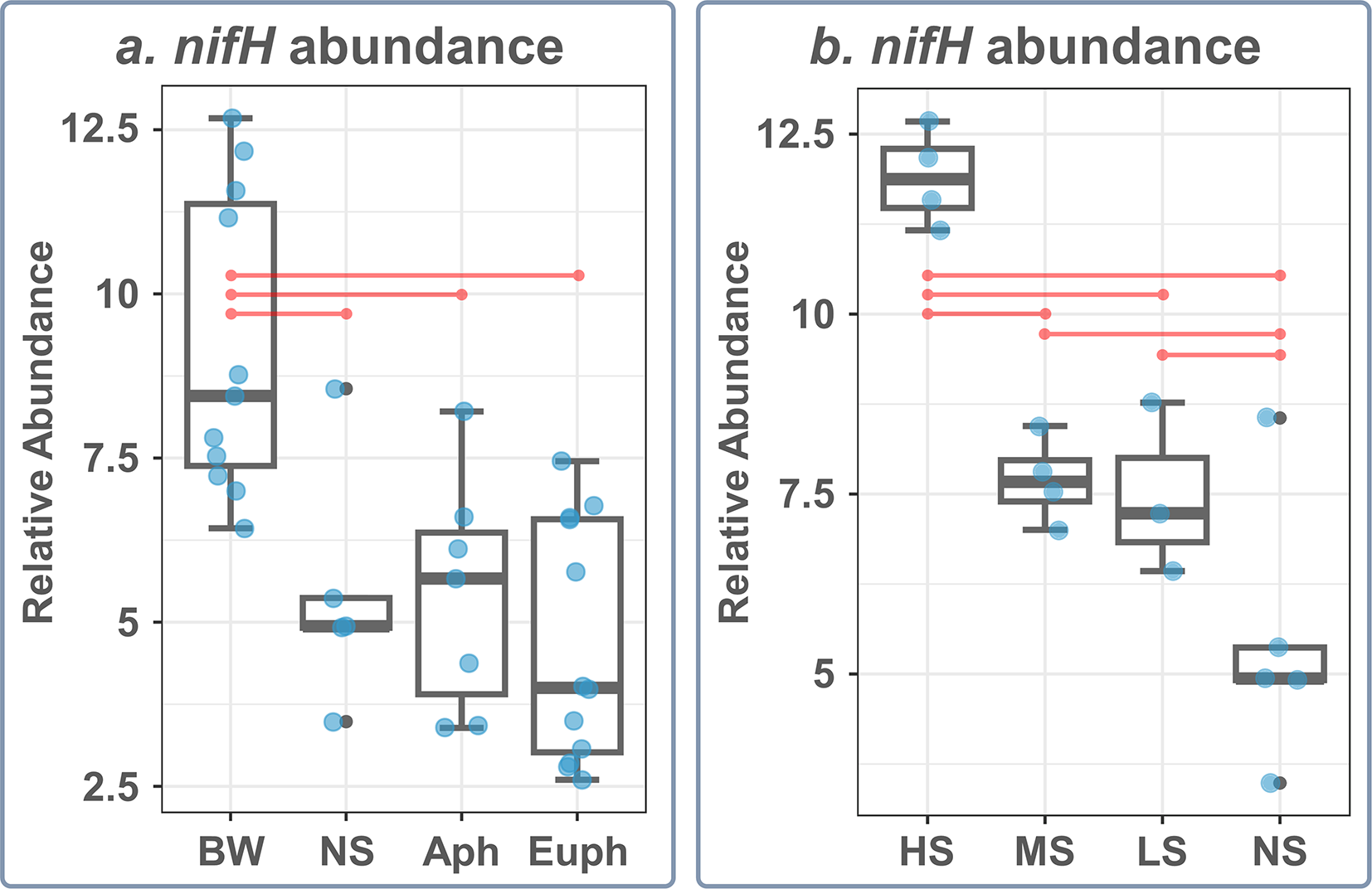

Supplement: Fig. S2 — Relative abundances of nifH sequences. [file msystems.00176-24-s0002.tif]

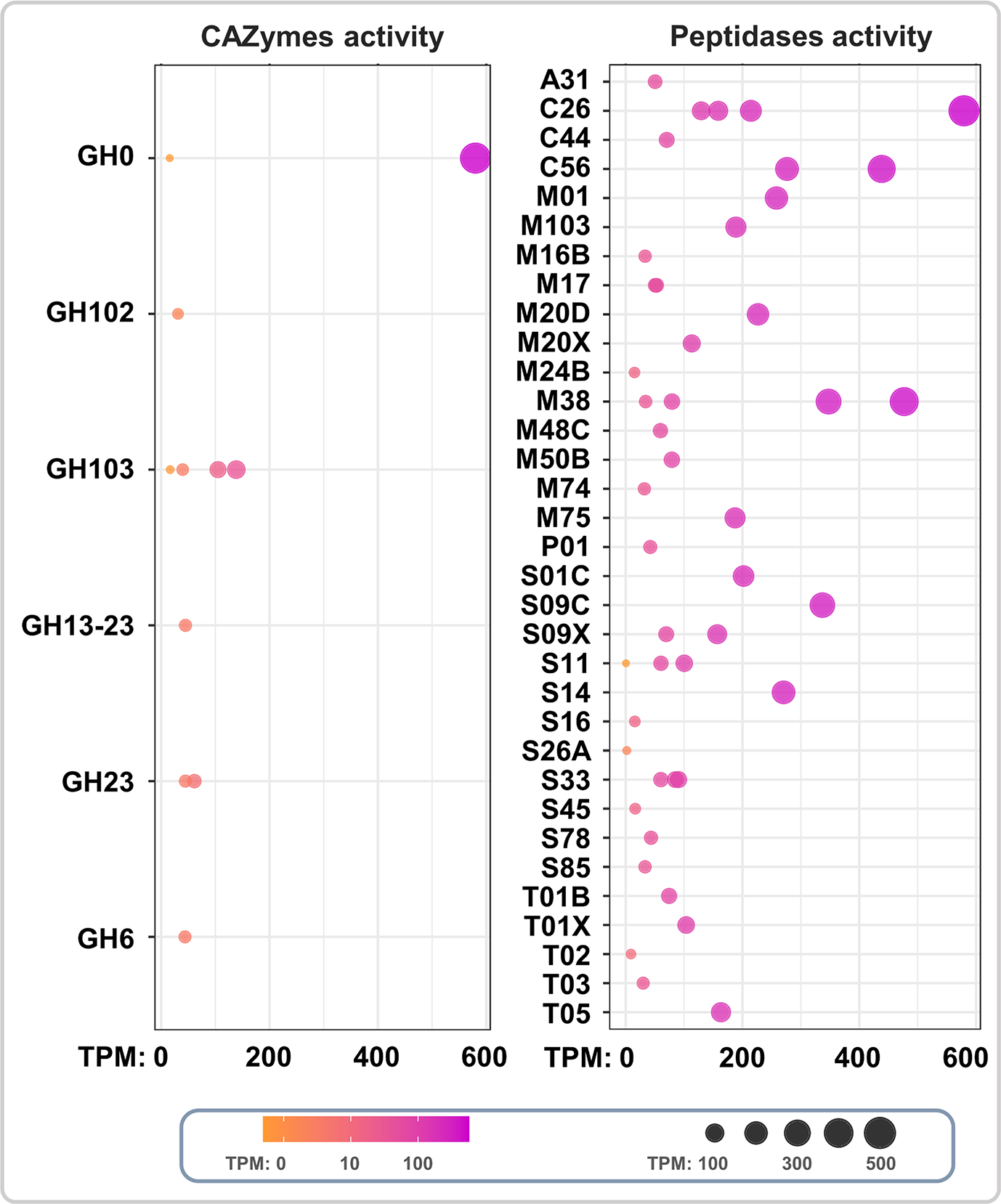

Supplement: Fig. S3 — Expression profiles of CAZymes and peptidases in Seep-BW-D1. [file msystems.00176-24-s0003.tif]

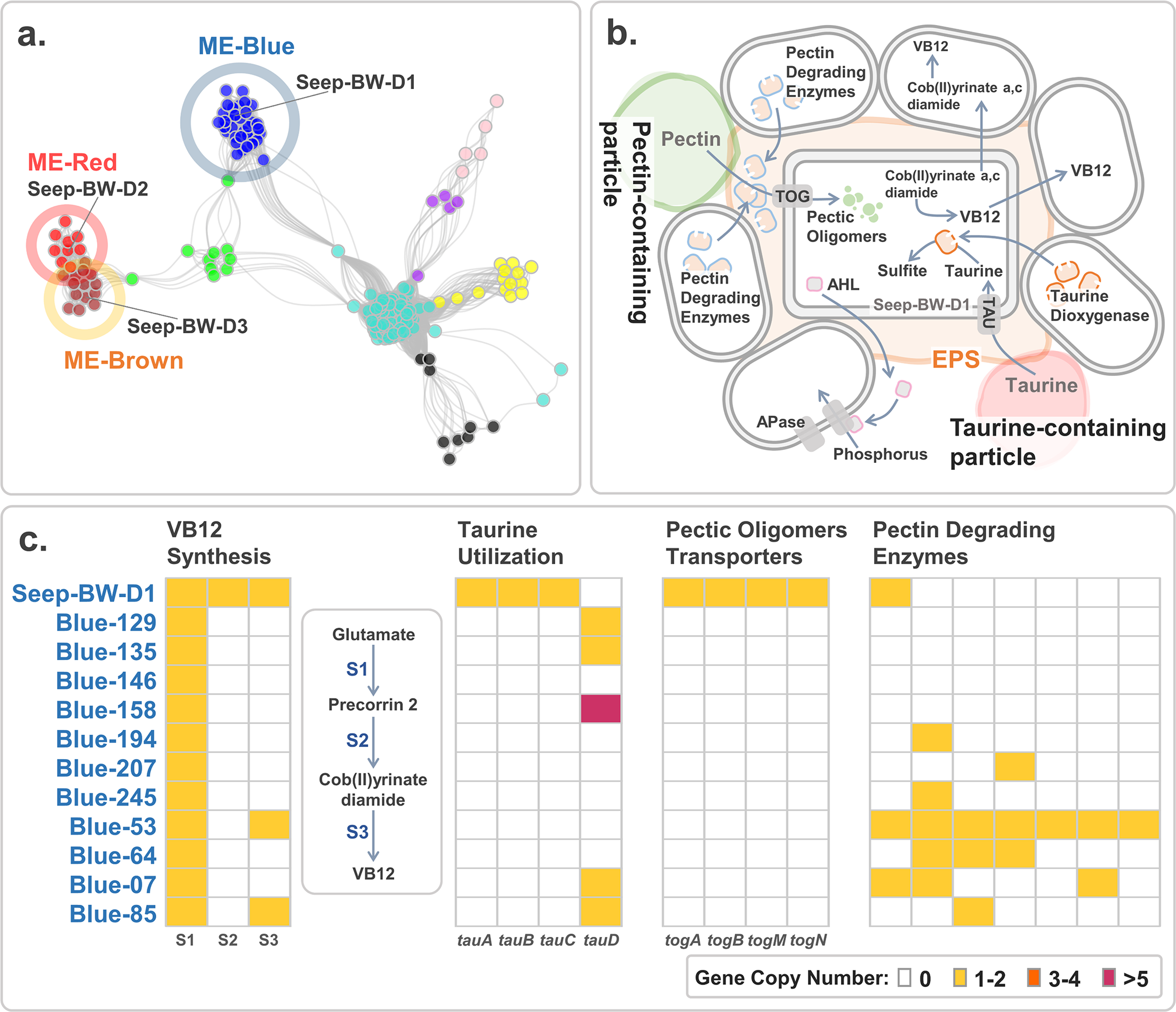

Supplement: Fig. S4 — WGCNA network analysis, potential interactions between Seep-BW-D1 and its co-occurring microbes, and copy number of genes involved in different functional categories among genomes in ME-Blue. [file msystems.00176-24-s0004.tif]
